# Supplementary material for: Ganglioside GD3 Regulates Inflammation and Epithelial-to-Mesenchymal Transition in Human Nasal Epithelial Cells
Source: Int J Mol Sci. 2024 Apr 5;25(7):4054. doi: 10.3390/ijms25074054 (PMC11012505; doi:10.3390/ijms25074054)
Supplement: Supplementary file 1 [file ijms-25-04054-s001.zip › ijms-2935726-supplementary.pdf]

Supplementary Materials: Ganglioside GD3 Regulates Inflammation and Epithelial-To-Mesenchymal Transition in Human Nasal Epithelial Cells

Ji Hyeon Hwang [1,2,†], Jae-Sung Ryu [1,†,‡], Jin Ok Yu [3], Young-Kug Choo [3,4], Jaeku Kang [2,5,*] and Jong-Yeup Kim [1,*]

1 Department of Otorhinolaryngology-Head and Neck Surgery, College of Medicine, Konyang University Hospital, Daejeon 35365, Republic of Korea; wgdrww5@gmail.com (J.H.H.); jsryu@kbiohealth.kr (J.-S.R.)
2 Department of Pharmacology, College of Medicine, Konyang University, Daejeon 35365, Republic of Korea
3 Department of Biological Science, College of Natural Sciences, Wonkwang University, Iksan 54538, Republic of Korea; yjo9703@naver.com (J.O.Y.); ykchoo@wku.ac.kr (Y.-K.C.)
4 Institute for Glycoscience, Wonkwang University, Iksan 54538, Republic of Korea
5 Priority Research Center, Myunggok Medical Research Institute, College of Medicine, Konyang University, Daejeon 35365, Republic of Korea
* Correspondence: jaeku@konyang.ac.kr (J.K.); jykim@kyuh.ac.kr (J.-Y.K.); Tel.: +82-42-600-9215 (J.K. & J.-Y.K.); Fax: +82-42-543-8959 (J.K. & J.-Y.K.)
† These authors contributed equally to this work.
‡ Current address: Division of Biodrug Evaluation, New Drug Development Center, Osong Medical Innovation Foundation (K-Bio Health), Cheongju 28160, Republic of Korea.

Supplementary Table S1. List of antibodies used in this study

Antibody	Company	Cat. No.	Application & Dilution
E-CADHERIN	Cell signaling	#3195	WB; 1:1,000 / IF; 1:200
N-CADHERIN	ABclonal	A19083	WB; 1:500 / IF; 1:100
SLUG	Cell signaling	#9585	WB; 1,000 / IF; 1:400
NK-κB p65	Cell signaling	#3039	WB; 1,000
β-ACTIN	Sigma-Aldrich	A5441	WB; 1:5,000
Histone H3	Cell signaling	#4499	WB; 1:2,000
Donkey anti-mouse IgG, Alexa 488	Thermo Fisher Scientific	A-21202	IF; 1:400
Donkey anti-rabbit IgG, Alexa 555	Thermo Fisher Scientific	A-31572	IF; 1:400
Goat anti-Mouse-HRP	Thermo Fisher Scientific	G-21040	WB; 1:10,000

Goat anti-Rabbit-HRP	Thermo Fisher Scientific	G-21324	WB; 1:10,000
DAPI	Sigma-Aldrich	D9542	IF; 30 nM

Supplementary Table S2. siRNA sequences for the knock-down of GD3 synthase (ST8Sia1)

Gene	Cat. No.	Sequence
siST8SIA1 #1	1021-1	CAC UUG GAC CAU GAC AGU A=tt(1-AS)<br>UAC UGU CAU GGU CCA AGU G=tt(1-AA)
siST8SIA1 #2	1021-2	ACA GCU UUG AUA CAC AGU A=tt(2-AS)<br>UAC UGU GUA UCA AAG CUG U=tt(2-AA)
siST8SIA1 #3	1021-3	CUG UAC UGG CGU GGA AGU U=tt(3-AS)<br>AAC UUC CAC GCC AGU ACA G=tt(3-AA)

Supplementary Table S3. Primer sequences used for qPCR analysis

Gene	Sequence (5`-3`)	Product size
IL-6	F: 5’- ACTCACCTCTTCAGAACGAATTG-3’<br>R: 5’- CCATCTTTGGAAGGTTCAGGTTG-3’	149 bp
IL-8	F: 5’- ACTGAGAGTGATTGAGAGTGGAC-3’<br>R: 5’- AACCCTCTGCACCCAGTTTTC-3’	112 bp
E-CAD	F: 5’- ATTTTTCCCTCGACACCCGAT-3’<br>R: 5’- TCCCAGGCGTAGACCAAGA-3’	109 bp
N-CAD	F: 5’- AGCCAACCTTAACTGAGGAGT-3’<br>R: 5’- GGCAAGTTGATTGGAGGGATG-3’	136 bp
SLUG	F: 5’- CTAACTGGACACACATACAGTG-3’<br>R: 5’- CTGAGGATCTCTGGTTGTGGT-3’	87 bp
MMP-9	F: 5’- AGACCTGGGCAGATTCCAAAC-3’<br>R: 5’- AGGCAAGTCTTCCGAGTAGT-3’	94 bp
ST8SIA1	F: 5’- CATGCGATGCAATCTCCCTC–3’<br>R: 5’- CTGGGATTAGCTGTCACTAACTG-3’	84 bp
CD73	F: 5’- AAGGACTGATCGAGCCACTC–3’	161 bp

	R: 5’- GGAAGTGTATCCAACGATTCCCA-3’	
CD90	F: 5’- ATGAAGGTCCTCTACTTATCCGC–3’<br>R: 5’- GCACTGTGACGTTCTGGGA-3’	112 bp
CD105	F: 5’- GCATCCTTCGTGGAGCTACC–3’<br>R: 5’- GAGGAGTGGTCTGGATCGG-3’	103 bp
β-ACTIN	F: 5’- TCCTCTCCCAAGTCCACACAGG-3’<br>R: 5’- GGGCACGAAGGCTCATCATTC-3’	131 bp

Supplementary Figure Legends

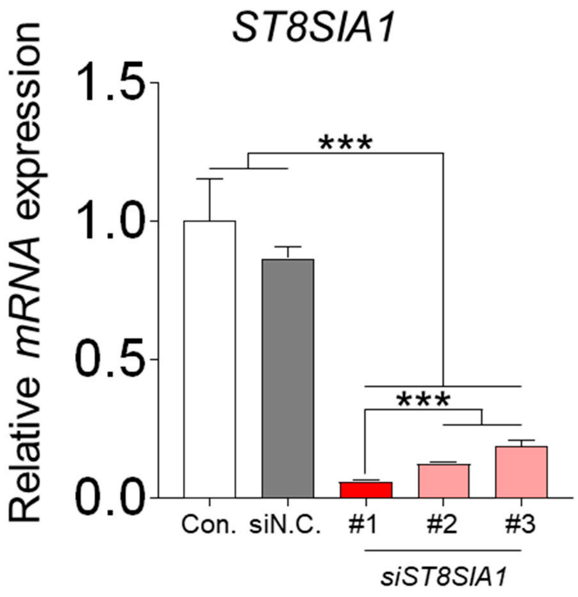

Figure S1. Knock-down efficiency of ST8Sia1 (GD3 synthase) expression in hNECs. hNECs were transfected with three different ST8Sia 1-targeting siRNAs (siST8Sia1 #1~#3). After 48 hours, siST8Sia1 #1 was found to significantly reduce the ST8Sia1 mRNA expression. mRNA expression levels were normalized to the housekeeping gene, β-Actin. The values shown are the mean ± SD ($n = 6$) and analyzed by Student`s t-test. $^{***}P < 0.001$.
